# Molluscicidal and Larvicidal Potency of *N*-Heterocylic Analogs against *Biomophalaria alexandrina* Snails and *Schistosoma mansoni* Larval Stages

**DOI:** 10.3390/pharmaceutics15041200

**Published:** 2023-04-10

**Authors:** Sherin K. Sheir, Elshaymaa I. Elmongy, Azza H. Mohamad, Gamalat Y. Osman, Shimaa E. Bendary, Abdullah A. S. Ahmed, Reem Binsuwaidan, Ibrahim El-Tantawy El-Sayed

**Affiliations:** 1 Zoology Department, Faculty of Science, Menoufia University, Shibin El Kom 32511, Egypt; sherin.sheir@yahoo.com (S.K.S.); azza_hassan_2006@yahoo.com (A.H.M.); dr.gyosman@yahoo.com (G.Y.O.); samshosalania52@yahoo.com (S.E.B.); 2 Department of Pharmaceutical Sciences, College of Pharmacy, Princess Nourah bint Abdulrahman University, P.O. Box 84428, Riyadh 11671, Saudi Arabia; rabinsuwaidan@pnu.edu.sa; 3 Chemistry Department, Faculty of Science, Menoufia University, Shibin El-Kom 32511, Egypt; chemist_abdullah_2009@yahoo.com (A.A.S.A.); ibrahimtantawy@yahoo.co.uk (I.E.-T.E.-S.)

**Keywords:** *Biomophalaria alexandrina*, *Cercaria*, *N*-heterocyclic analogs, SEM, biological aspects

## Abstract

This work describes the synthesis of quinoline-based *N*--heterocyclic arenes and their biological evaluation as molluscicides against adult *Biomophalaria alexandrina* snails as well as larvicides against *Schistosoma mansoni* larvae (miracidia and cercariae). Molecular docking studies were demonstrated to investigate their affinity for cysteine protease protein as an interesting target for antiparasitics. Compound **AEAN** showed the best docking results followed by **APAN** in comparison to the co-crystallized ligand D1R reflected by their binding affinities and RMSD values. The egg production, hatchability of *B. alexandrina* snails and ultrastructural topography of *S. mansoni* cercariae using SEM were assessed. Biological evaluations (hatchability and egg-laying capacity) revealed that the quinoline hydrochloride salt **CAAQ** was the most effective compound against adult *B. alexandrina* snails, whereas the indolo-quinoline derivative **APAN** had the most efficiency against miracidia, and the acridinyl derivative **AEAA** was the most effective against cercariae and caused 100% mortality. **CAAQ** and **AEAA** were found to modulate the biological responses of *B. alexandrina* snails with/without *S. mansoni* infection and larval stages that will affect *S. mansoni* infection. **AEAA** caused deleterious morphological effects on cercariae. **CAAQ** caused inhibition in the number of eggs/snail/week and reduced reproductive rate to 43.8% in all the experimental groups. **CAAQ** and **AEAA** can be recommended as an effective molluscicide of plant origin for the control program of schistosomiasis.

## 1. Introduction

Schistosomiasis (Bilharziasis) is a parasitic disease caused by trematode blood flukes of genus Schistosoma. Assessments show that at least 236.60 million people needed preventive treatment for schistosomiasis in 2019, out of which more than 105.40 million people were reported to have been treated [1]. Biomophalaria snails as the intermediate host of *S. mansoni* have great medical importance. One of the methods for controlling Schistosomiasis is to break the life cycle of the parasite by combating its snail host. King and Bertsch [2] mentioned that control is a key intervention to achieving the WHO goal of Schistosomiasis eradication. Coustau and Théron [3] define compatibility as a characteristic of a host–parasite system, where the parasite species have the ability to obtain transmission using the host species and establishing infection. Many investigations were conducted to study the effects of different molluscicides of plant origin at low concentrations on the biological aspects of the snails and the larval stages of *S. mansoni* [4,5,6,7].

**Figure 1:**
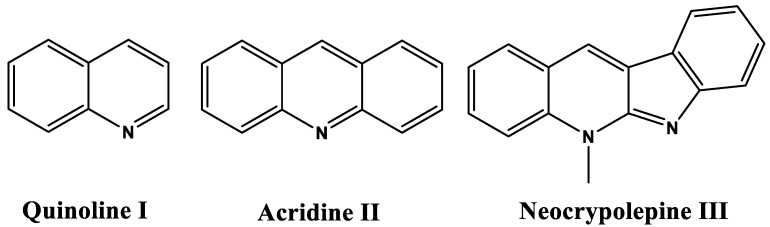
Naturally occurring *N*-heterocyclic arenes.

Naturally occurring quinoline I is one of the pharmaceutical cores used in drug discovery and development, Figure 1. This *N*-heterocyclic pharmaceutical core has potent biological importance as antimicrobial [8], antiviral [9], antioxidant [10], anti-inflammatory [11] and anticancer agents [12,13]. Recent literatures reported the potency of quinolone-based drugs as anti-schistosomiasis [14]. On the other hand, natural or synthetic tricyclic acridine II c.f. Figure 1 gains great interest in medicinal chemistry owing to its biological importance [15,16,17,18], Figure 1. Many hybridizations were applied on acridine with other pharmaceutical cores with potent biological activities and synergistic effects against different diseases, especially against schistosomiasis [19,20]. It is noteworthy to mention that the promising indoloquinolines isolated from the roots of the climbing shrub of *Cryptolepis sanguinolenta* have tunable activities against different diseases [21]. Among thirteen alkaloids presenting in *Cryptolepis sanguinolenta*, neocryptolepine III has various biological activities as anticancer [22], antimalarial [23], antimicrobial [21], anti-inflammatory [24] and anti-schistosomiasis agents [25,26], Figure 1. In our previous study for neocryptolipine-like derivatives derived from quinoline, we have evaluated the toxicity towards the normal human MRC5 and L6 cells, and we did not observe any significant toxicity [23,24]. In light of the aforementioned, we were encouraged to make structural modifications aiming to evaluate the molluscicidal activity of natural-based analogues derived from quinoline against *B. alexandrina* snail, the intermediate host of *S. mansoni* and its larval stages (miracidia and cercariae) as well as to explore its potential use as a safe molluscicide and its impact on the biological aspects of the snail.

## 2. Materials and Methods

### 2.1. Chemistry 

NMR spectroscopic analyses were carried out at Zagazig University with the Bruker magnet system 400, 54 Ascend/R (Houston, TX, USA) 400 MHz with 100 MHz for ^1^H NMR and ^13^C NMR, respectively. All starting materials such as 4,7-dichloroquinoline, Diamines were purchased from Sigma Aldrich, Saint Louis, MO, USA. The afforded products used here in this study were prepared according to reported methods and showed spectroscopic data consistent with reported ones [22,23,25,27,28]. The synthesis of the new hydrochloride salt of N1-(7-chloroquinolin-4-yl) ethane-1,2-diamine hydrochloride 4 was prepared and characterized as follows:

An excessive amount of ethylene diamine 2 was added to 4,7-dichloroquinoline 1 under reflux condition for 2 h to obtain free amine product 3 with good yield. The mixture was poured into ice water, filtered off and dried to afford the free amine 3. In the next step, 1 M of hydrochloric acid was added to the ethanolic solution of free amine 3, and the pH was adjusted till the formation of the hydrochloride salt of free amine 4 with good yield. The formation of hydrochloride salt was characterized by ^1^H NMR and ^13^C NMR (Supplementary Materials). Off white solid, yield (88%), ^1^H NMR (D_2_O-400 MHz) δ: 3.45 (t, 2H, CH_2_, *J* = 8 Hz), 3.99 (t, 2H, CH_2_, *J* = 8 Hz), 6.89 (d, 1H, CH_Ar_, *J* = 8 Hz), 7.64 (d, 1H, CH_Ar_, *J* = 8 Hz), 7.83 (s, 1H, CH_Ar_), 8.13 (d, 1H, CH_Ar_, *J* = 8 Hz), 8.40 (d, 1H, CH_Ar_ = N, *J* = 8 Hz). ^13^C NMR (D_2_O-101 MHz) δ: 37.44, 40.32, 98.49, 115.36, 119.14, 124.08, 127.80, 137.94, 139.58, 142.74, 156.24.

### 2.2. Modeling Studies

Molecular docking studies were performed using MOE (molecular operating environment, Chemical Computing Group ULC, 1010 Sherbooke St. West, Suite #910, Montreal, QC, Canada). Cysteine protease protein was downloaded for modeling study from protein data bank (pdb: 2P7U) [29,30]. Protein and ligand optimizations were applied by calculating partial charges, 3D protonation, correction of the missed strands then saved as mol. file after energy minimization as reported [31]. Applying induced fit as a docking protocol, docking site was selected at the ligand site for PDB: 2P7U alpha spheres were selected as a guide to the placement with 5 A. Docking site was at dummy site for PDB: 1CJL as it was downloaded as apoprotein. Browsed database of the investigated compound in mdb format and pharmacophore annotations were excluded. The gradient for energy was minimized to 0.05, and force field was chosen as MMFF94X by the system default [32]. 

### 2.3. Biological Study

#### 2.3.1. Snails

The experimental snails used in the present study were adult *B. alexandrina* (shell diameter: 8–10 mm). The snails were obtained from Schistosome Biological Supply Program (SBSP), Theodor Bilharz Research Institute, Giza, Egypt.

#### 2.3.2. Miracidia

Eggs of *S. mansoni* were obtained from Schistosome Biological Supply Program (SBSP), Theodor Bilharz Research Institute, Giza, Egypt. Eggs were left in clean dechlorinated tap water for hatching under the light. Freshly hatched miracidia were used in infection tests and bioassays.

#### 2.3.3. Cercariae

Cercariae were shed under illumination from the infected snails obtained from (SBSP), Theodor Bilharz Research Institute, Giza, Egypt.

#### 2.3.4. Snails Breeding and Maintenance

Adult snails were kept in plastic aquaria (4 L capacity) containing dechlorinated tap water. Egg masses were collected from the small foam pieces placed on the water surface of the aquaria and kept in smaller plastic aquaria of 2 L capacity containing dechlorinated tap water [33]. Hatched snails were transferred with a fine brush to another aquarium where they were fed algae, Nostoc commune, chalk as a calcium supplement and aseptic soil [34].

After reaching a 2–4 mm shell diameter, they were fed lettuce and tetramine (fish food). The water in the aquaria was regularly changed twice a week.

#### 2.3.5. Snails Infection and Larval Stages Maintenance

Miracidia of *S. mansoni* were hatched under illumination from eggs isolated from homogenized liver and intestine of 6–8 weeks infected CD1 mice from SBSP [35,36]. The miracidia were collected from the side-arm flask by a Pasteur pipette. Mature snails (8–11 mm shell diameter) were exposed individually to 5–7 miracidia in a glass test tube filled with 2 mL dechlorinated tap water for 2 h [37].

### 2.4. Toxicity Test

#### 2.4.1. Molluscicidal Activity

Six heteroarene derivatives **CAAQ** (**4a**), **APACQ** (**4b**), **AEAA** (**4c**), **APAA** (**4d**), **AEAN** (**7a**) and **APAN** (**7b**) were tested against adult *B. alexandrina* snails. A stock solution of 1000 ppm was freshly prepared based on *w*/*v* using dechlorinated tap water (pH 7.5–7.7). The quality control (toxicity) experiment concentrations were chosen using a series of concentrations (1000, 750, 500, 250, 100, 55, 50, 45, 40, 35, 30, 25 and 20 ppm) and were prepared to allow the computation of LC_50_ and LC_90_ values. Three replicates were used, each of 10 snails being immersed in one liter of each tested concentration. The exposure period was 96 h at room temperature. Three replicates of control snails were kept under the same experimental conditions in dechlorinated tap water without treatment. Dead snails were regularly counted and removed from each group. LC_50_ and LC_90_ were computed using Statgraphics Centurion XVI as the following formula:

Tested compound concentration = EXP (3.18823 + 0.277043 × SQRT (mortality))

#### 2.4.2. Larvicidal Activity

##### Miracidicidal Activity

In vitro exposure of miracidia (obtained from SBSP) was done. Three heteroarene derivatives (**CAAQ**; **AEAA** and **APAN**) were tested against miracidia after 90 minutes of exposure. A series of concentrations (150, 100, 75, 50, 25, 10 and 5 ppm) were prepared to allow the computation of LC_50_ and LC_90_ values. LC_50_ and LC_90_ were computed using Statgraphics Centurion XVI following this formula:

Concentrations = EXP (1.08971 + 1.14411 × SQRT (mortality))

##### Cercaricidal Activity

Six heteroarene derivatives (**CAAQ**; **AEAA**; **APACQ**; **AEAN**; **APAN** and **APAA**) were tested against cercariae. Cercariae were shed from the infected snails (obtained from SBSP) and were transferred to Petri dishes containing the tested concentrations (300, 250, 200, 150, 100, 50, 40, 30, 20, 10 and 5 ppm) and were prepared to allow the computation of LC_50_ and LC_90_ values. Three replicates for each experimental material on 10–15 *S. mansoni* cercariae were done. Dead (motionless) cercariae were recorded at time intervals of 5–90 min, using a binuclear dissecting microscope. LC_50_ and LC_90_ were computed using Statgraphics Centurion XVI following this formula:

Concentrations = EXP (−2.59437 + 0.750867 × SQRT (mortality))

### 2.5. Scanning Electron Microscope Study of S. mansoni Cercariae

Cercariae were exposed in vitro to 50 ppm of **CAAQ** or **AEAA** for 90 min. They were fixed in 2.5% glutaraldehyde and 2% paraformaldehyde in 0.1 M cacodylate buffer (pH 7.4). The surface ultrastructure of cercariae was examined and photographed using a Scanning Electron Microscope (A JEOL JSM-5300 at the voltage of (20–25) KV, Tokyo, Japan) following the routine method with magnification from 450 to 4500 X, at the Faculty of Science, Alexandria University, Bab Sharqi, Egypt [38].

### 2.6. Experimental Design

Sub-lethal concentrations (0.25 and 1 ppm) of **CAAQ** were used in this study. A total of 540 mature snails (8–11 mm shell diameter) were divided into six groups. The 1st group was kept as the control group (neither exposed nor infected group). The 2nd group was exposed to 1 ppm of **CAAQ**, whereas the 3rd group was infected with *S. mansoni* (infected/positive control). The 4th group was exposed to 1 ppm of **CAAQ** and infected with *S. mansoni* (exposed + infected group). Then, the 5th group was exposed to 0.25 ppm of **CAAQ**. Finally, the 6th group was exposed to both 0.25 ppm of **CAAQ** and *S. mansoni* infection (exposed + infected group). Snails (30 snails/replicate and 90 snails/group) were immersed in 1000 mL of water treated with the experimental concentrations in plastic containers of 4 L capacity. Exposed water was regularly changed with freshly prepared ones twice a week. The experiment lasted for 2 weeks, and the egg-laying capacity, hatchability and egg abnormality were recorded daily.

#### 2.6.1. Egg-Laying Capacity

Egg-laying capacity was determined by counting the number of egg masses/snail/week under a stereomicroscope (Optika, Bergamo, Italy) after 24 h, one week and two weeks. The egg-laying capacity was expressed as the number of eggs/snail/week (MX) according to [39]. Egg abnormalities were recorded and photographed by Optika digital camera, Italy.

#### 2.6.2. Hatchability of Eggs of *B. alexandrina* In Vitro

Three replicates of egg masses each of about 30 ± 5 eggs were added to each experimental concentration (0.25 and 1 ppm) and the control group was maintained in dechlorinated tap water [40]. Egg masses laid on foam pieces were collected from healthy *B. alexandrina* snails. Egg masses were exposed to 20 mL of tested solutions in Petri dishes till hatching. The number of normal viable eggs and hatched embryos were recorded [33]. At the end of the experiment, the percentage of hatchability was calculated by dividing the number of hatched embryos at the end of the experimental period by the total number of eggs at the beginning of the experiment. 

#### 2.6.3. Statistical Analysis

All data sets were analyzed using Statgraphics Centurion XVI (Stat-Point Technologies Inc., Warrenton, VA, USA). The statistical analysis was carried out by one-way analysis of variance (ANOVA) under the effect of exposure and time in experimental studies. The exposed groups were compared with each other and with the control groups. Data were considered significant when *p* ≤ 0.05. LC values were calculated using simple regression relationship between the mortality of the organisms and the concentrations of the related compounds.

## 3. Results

### 3.1. Chemistry 

**Scheme 1:**
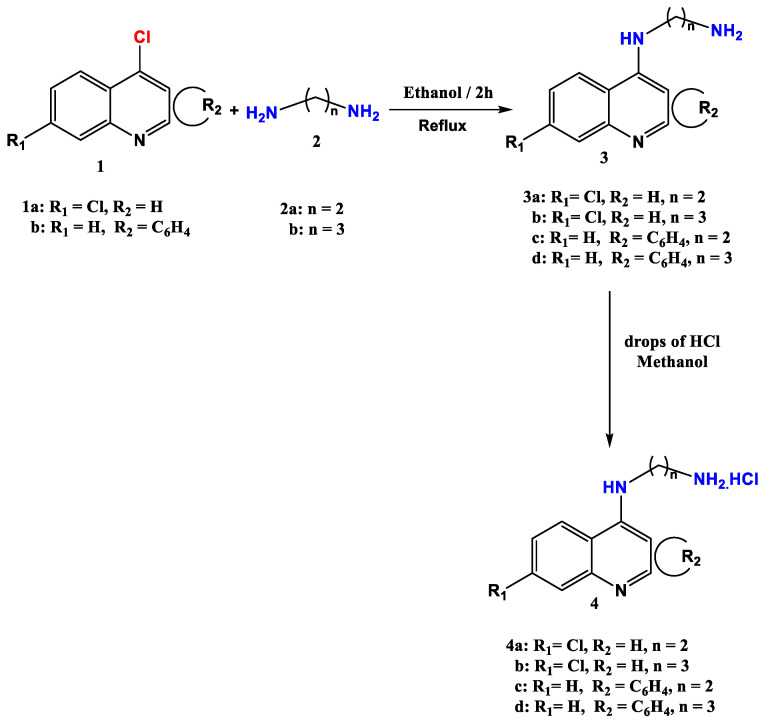
Synthesis of amino alkylamino acridine/quinoline derivatives **4a**–**d**.

**Scheme 2:**
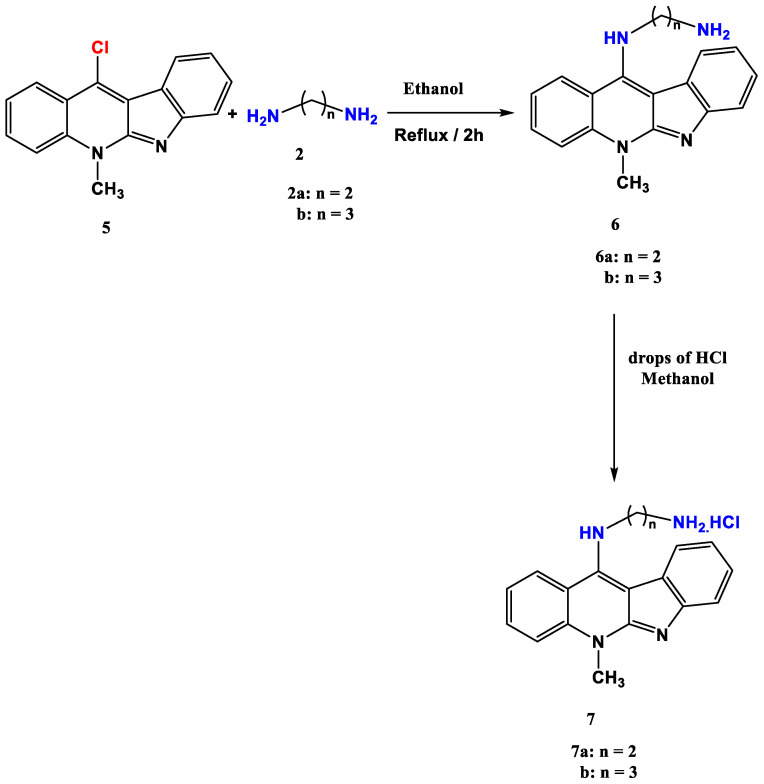
Synthesis of 11-aminoalkylamino neocryptolepine derivatives **7a**,**b**.

Synthesis of amino alkylamino derivatives bearing quinoline and acridine cores was achieved by nucleophilic substitution reaction (SN_Ar_) through the addition of 4,7-dichloroquinoline **1a** or 9-chloroacridine **1b** to an excessive number of diamines **2a** and **2b** (3 mmol) in presence of (3 mL) of DMF to obtain the free amine derivatives of quinoline and acridine containing two and three carbon spacers **3a**–**d** in good yields. Further acidification was carried out on free amines of quinoline and acridine derivatives dissolved in methanol by using drops of 1 M of HCl till the formation of the hydrochloride salt of these derivatives **CAAQ** (**4a**), **APACQ** (**4b**), **AEAA** (**4c**) and **APAA** (**4d**) as reported in literatures [22,28], Scheme 1. On the same trend, the formation of hydrochloride salts of 11-amino alkylamino neocryptolepine **6a**,**b** was obtained by reaction of (1 mmol) of the starting 11-chloroneocryptolepine **4** with an excess amount of ethylene diamine **2a** or 1,3-diamino propane **2b** (3 mmol) ethanol (3 mL) to afford **AEAN** (**7a**) and **APAN** (**7b**) in good yields. Finally, the corresponding salts of the free amine bases **7a** and **7b** were prepared by addition of few drops of 1 M of HCl to methanolic solution of **6a** and **6b** with pH control as illustrated in Scheme 2, as reported in literatures [23,25,27]. 

### 3.2. Modeling Studies 

**Figure 2:**
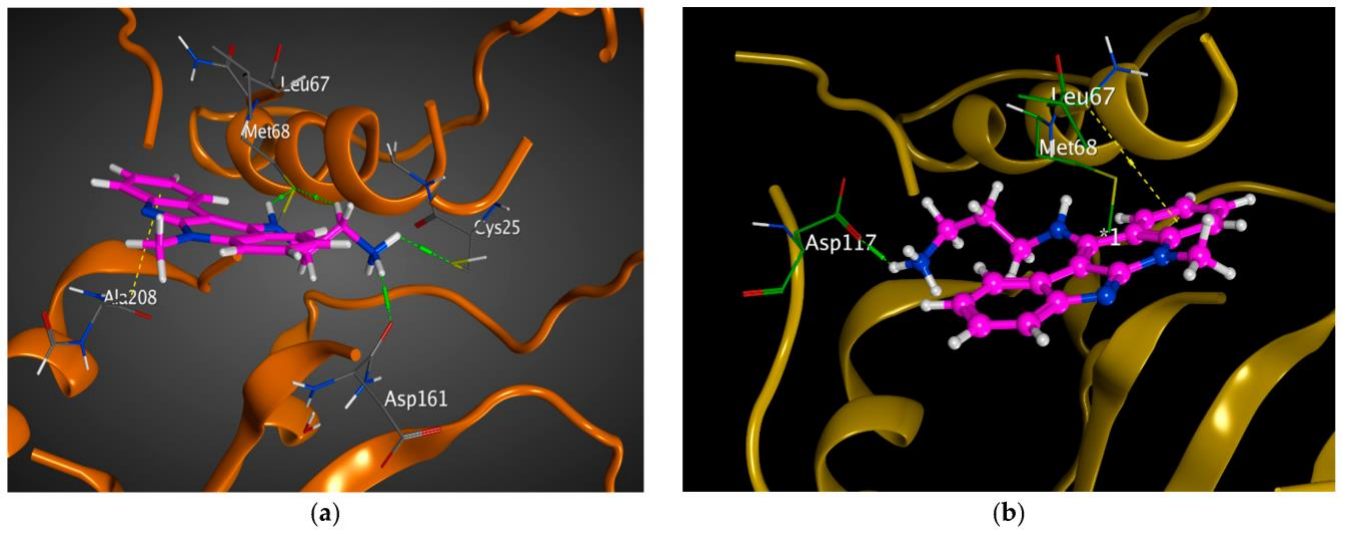
Chemical bonding and aminoacid interactions in the active site of cysteine protease (**a**) **AEAN**; (**b**) **APAN**, where *1 is the position of the co-crystalized ligand (D1R).

Molecular docking studies were demonstrated on six prepared compounds to investigate their affinity for cysteine protease protein. Cysteine protease protein was downloaded from protein data bank PDB code: 2P7U. The co-crystalized ligand (D1R) was redocked in 2P7U in a step for results verification. All the tested compounds showed promising affinities to cysteine protease ranging from −5.9386 to −4.8697 and the root mean square deviation (RMSD) values range from 1.4522 to 2.0628 in comparison to the co-crystallized ligand D1R that showed affinity of −5.678 and RMSD value of 1.4811. Compound **AEAN** showed the best docking results followed by **APAN** with binding affinities −5.9386 and −5.6194 and RMSD values of 1.4522 and 1.7254, respectively. Tabulated below in Table 1 are the binding affinity scores (s) and RMSD results, in addition to the amino acid residues and the type of bonding while interacting with the downloaded protein. Chemical bonding and amino acid residues involved in the interaction between the downloaded protein and compounds with the top results **AEAN** and **APAN** are illustrated in Figure 2.

### 3.3. The Toxic Effects of Synthesized N-Heteroarene Derivatives 

#### 3.3.1. Molluscicidal Activity

**Figure 3:**
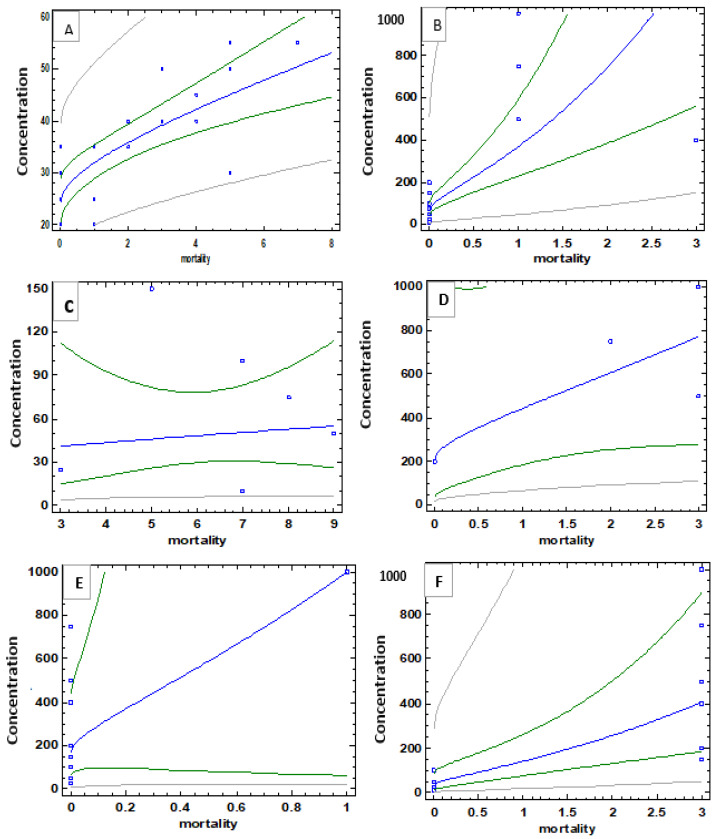
Effect of the molluscicidal activity of six heteroarene derivatives (**A**) **CAAQ**; (**B**) **AEAA**; (**C**) **APACQ**; (**D**) **AEAN**; (**E**) **APAN** and (**F**) **APAA** against adult *B. alexandrina* snails after 96 h of exposure.

The molluscicidal activity of six heteroarene derivatives (**CAAQ**; **AEAA**; **APACQ**; **AEAN**; **APAN** and **APAA**) against adult *B. alexandrina* snails were tested after 96 h of exposure under laboratory conditions. The results showed that **CAAQ** was the best molluscicide against *B. alexandrina* adult snails as it was completely soluble in water, while the other derivatives were not completely soluble. The results showed that LC_50_ and LC_90_ values of **CAAQ** were 171.95 and 335.79 ppm, respectively, with slope function values of 0.28. LC_50_ and LC_90_ values of **AEAA** were 1084.7 × 10^4^ and 650.5 × 10^6^ ppm, respectively, with a slope function value of 1.7. For **APACQ**, LC_50_ and LC_90_ values were 230.7 × 10^3^ and 380.3 × 10^4^ ppm, respectively, with a slope function value of 1.16. LC_50_ and LC_90_ values of **AEAN** were 895.8 × 10^3^ and 291.7 × 10^5^ ppm, respectively, with slope function value of 1.44. For **APAN**, LC_50_ and LC_90_ values were 438.2 × 10^2^ and 272.6 × 10^3^ ppm, respectively, with slope function value of 0.76. LC_50_ and LC_90_ values of APAA were 553.2 × 10^5^ and 426.7 × 10^7^ ppm, respectively, with slope function value of 1.8 as depicted in Figure 3. 

#### 3.3.2. Larvicidal Activity

**Figure 4:**
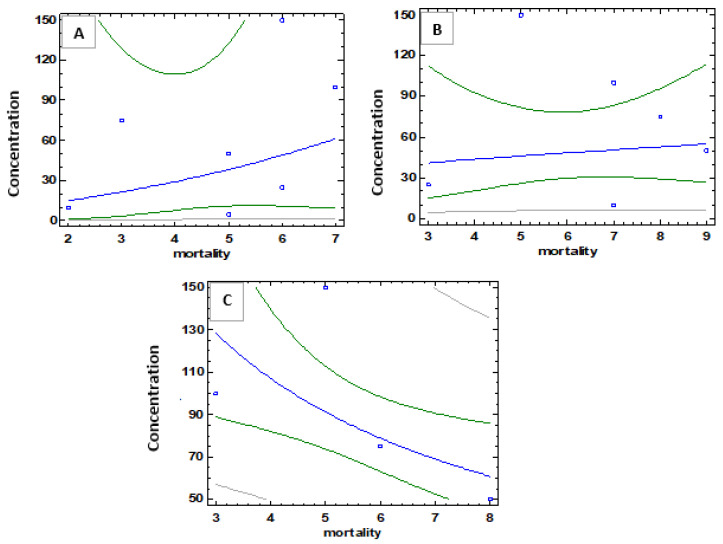
Effect of miracidial activity of three heteroarene derivatives (**A**) **CAAQ**; (**B**) **AEAA** and (**C**) **APAN** against miracidia of *S. mansoni* after 90 min of exposure.

For the miracicidal activity, three compounds (**CAAQ**; **AEAA** and **APAN**) were tested against miracidia after 90 min of exposure, as they were more soluble in water than **APACQ**, **AEAN** and **APAA**. They could not be applied because they did not dissolve properly, making a suspended solution giving an unclear field under the microscope and made it so difficult to examine and differentiate the live and the dead miracidia even after the filtration of the solutions. There was an inverse relationship between concentration and time of exposure against miracidia as shown in Figure 4. The results showed that **APAN** had the most efficiency against miracidia. The results showed that LC_50_ and LC_90_ values of **APAN** were 3.4 and 0.7 ppm, respectively, with slope function value of −0.68. For **CAAQ**, LC_50_ and LC_90_ values were 969.9 × 10 and 153.8 × 10^3^ ppm, respectively, with slope function value of 1.14. LC_50_ and LC_90_ values of **AEAA** were 140.1 and 244.3 ppm, respectively, with a slope function value of 0.23.

**Figure 5:**
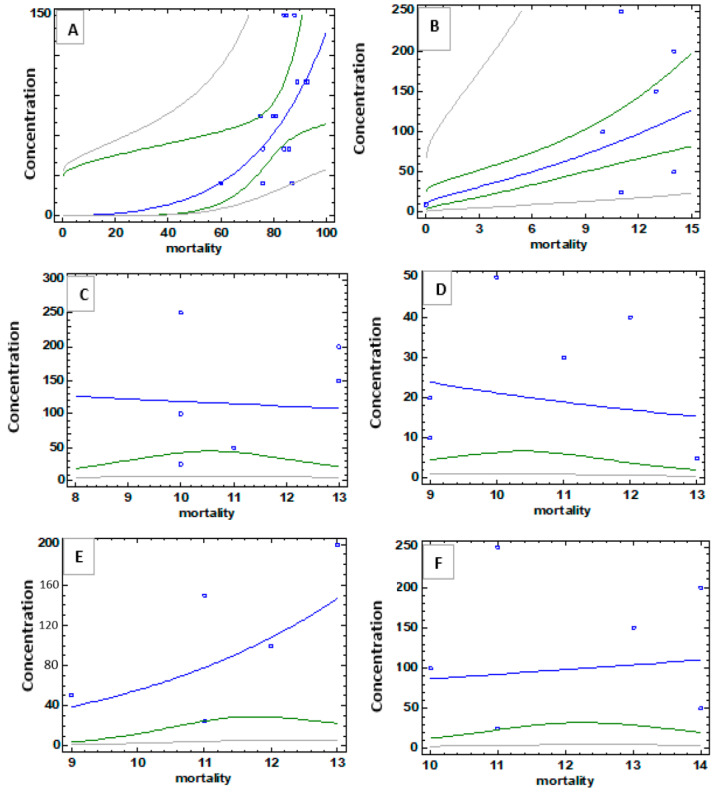
Effect of the cercarial activity of six heteroarene derivatives (**A**) **CAAQ**; (**B**) **AEAA**; (**C**) **APACQ**; (**D**) **AEAN**; (**E**) **APAN** and (**F**) **APAA** against cercariae of *S. mansoni* after 90 min of exposure.

For the cercaricidal activity, compounds (**CAAQ**; **AEAA**; **APACQ**; **AEAN**; **APAN** and **APAA**) were tested for the larvicidal activity against cercariae after 90 min of exposure. The most effective compound against cercariae was **AEAA** as it recorded 100% mortality at the lowest concentration of 25 ppm after two and a half hours. The results showed that LC_50_ and LC_90_ values of **AEAA** were 1.25 and 0.22 ppm, respectively, with a slope function value of −0.73. For **CAAQ**, LC_50_ and LC_90_ values were 15.1 and 92.7 ppm, respectively, with a slope function value of 0.75. For **APACQ**, LC_50_ and LC_90_ values were 54.2 and 33.5 ppm, respectively, with a slope function value of −0.20. LC_50_ and LC_90_ values of **AEAN** were 9.3 and 0.4 ppm, respectively, with a slope function value of −1.34. For **APAN**, LC_50_ and LC_90_ values were 287.8 × 10^3^ and 567.8 × 10^5^ ppm, respectively, with a slope function value of 2.19. LC_50_ and LC_90_ values of **APAA** were 440.6 and 120.4 × 10 ppm, respectively, with a slope function value of 0.42 as reported in Figure 5.

#### 3.3.3. Effect of **CAAQ** on Biological Activities of *B. alexandrina* Snails

##### Egg-Laying Capacity

The egg-laying capacity of *B. alexandrina* snails was markedly affected as a result of **CAAQ** exposure and/or *S. mansoni* infection. **CAAQ** exposure had markedly affected the number of egg masses/snail/week and eggs/snail/week during the duration of the experiment (2 weeks, Table 2).

After 24 h of exposure, the number of egg masses/snail/week for the exposed snails to 0.25 ppm and 1 ppm were 0.04 ± 0.05 and 0.07 ± 0.09, respectively. The number of egg masses/snail/week for infected-exposed snails to 0.25 ppm and 1 ppm were 0.08 ± 0.02 and 0.18 ± 0.02, respectively, when compared to 11.0 ± 0.05 and 0.18 ± 0.08 of control and infected control groups, respectively (*p* ≥ 0.05). After one week of exposure (*p* ≤ 0.002), the number of egg masses/snail/week for exposed snails to 0.25 ppm and 1 ppm were 0.16 ± 0.02 and 0.4 ± 0.28, respectively. The number of egg masses/snail/week for infected-exposed snails to 0.25 ppm and 1 ppm were 0.16 ± 0.02 and 0.09 ± 0.08, respectively, when compared to 2.80 ± 0.38 and 1.97 ± 0.46 of control and infected control groups, respectively. After two weeks of exposure, the number of egg masses/snail/week of exposed snails to 0.25 ppm and 1 ppm were 0.17 ± 0.04 and 0.06 ± 0.05, respectively. The number of egg masses/snail/week of the infected-exposed snails to 0.25 ppm and 1 ppm were 0.18 ± 0.05 and 0.09 ± 0.12, respectively, when compared to 2.67 ± 0.33 and 1.96 ± 0.47 of control and infected control groups, respectively (*p* ≤ 0.000).

After 24 h of exposure, the number of eggs/snail/week for exposed snails to 0.25 ppm and 1 ppm were 0.89 ± 1.13 and 1.38 ± 1.77, and the number of eggs/snail/week for infected-exposed snails to 0.25 ppm and 1 ppm were 1.03 ± 0.35 and 1.79 ± 0.25 when compared to 2.07 ± 0.90 and 2.34 ± 0.80 of control and infected control groups, respectively (*p* ≥ 0.05). After one week of exposure, the number of eggs/snail/week for exposed snails to 0.25 ppm and 1 ppm were 2.23 ± 0.60 and 9.70 ± 6.49, and the number of eggs/snail/week for infected-exposed snails to 0.25 ppm and 1 ppm were 1.57 ± 0.12 and 0.92 ± 0.83 when compared to 54.69 ± 6.31 and 28.37 ± 5.87 of control and infected control groups, respectively (*p* ≤ 0.002). After two weeks of exposure, the number of eggs/snail/week for exposed snails to 0.25 ppm and 1 ppm were 2.25 ± 0.49 and 0.91 ± 0.81, and the number of eggs/snail/week for infected-exposed snails to 0.25 ppm and 1 ppm were 2.10 ± 1.19 and 1.45 ± 1.93 when compared to 46.26 ± 8.48 and 28.10 ± 11.59 of the control and the infected control groups, respectively (*p* ≥ 0.05).

**Figure 6:**
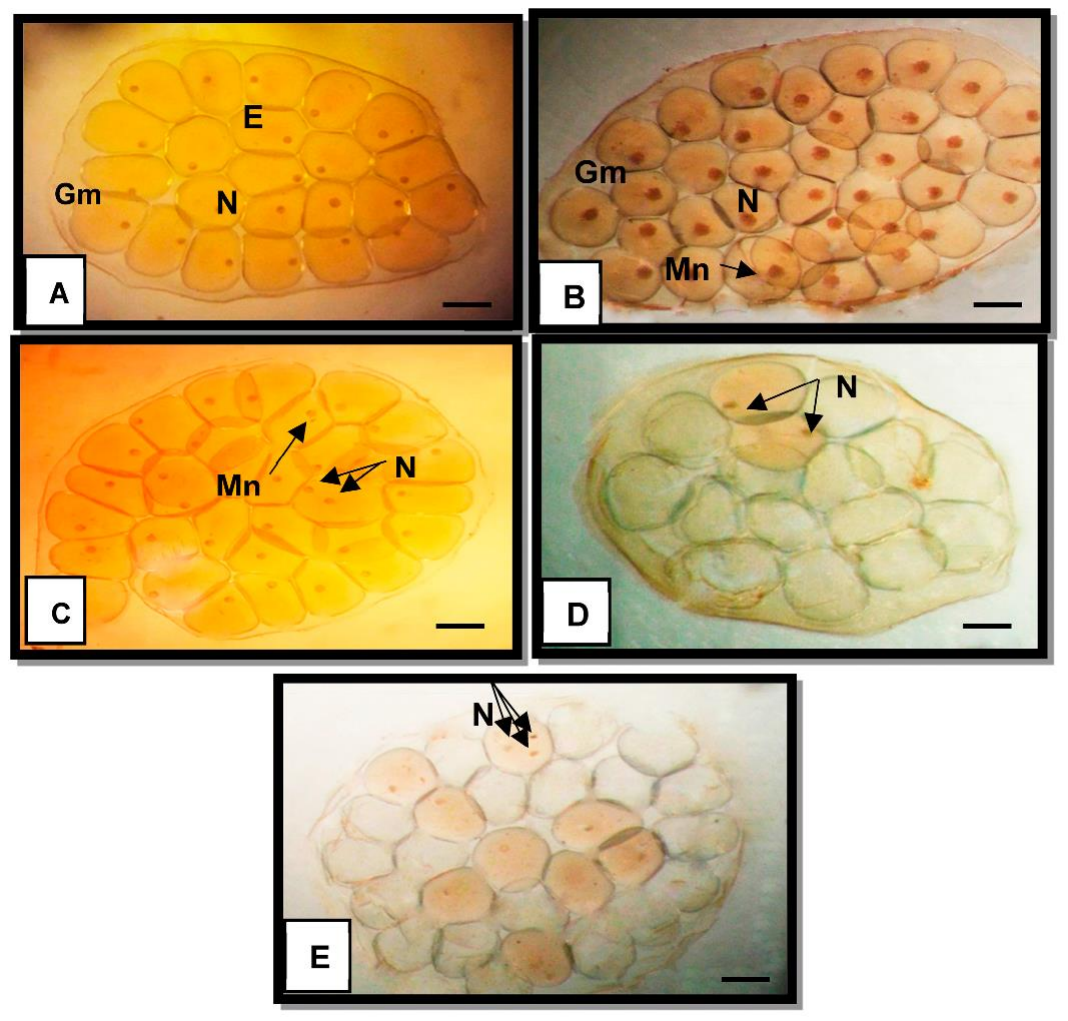
Light photomicrographs of *B. alexandrina* egg masses: (**A**,**B**) egg masses of the control group; (**C**) egg masses laid by *B. alexandrina* snails exposed to sub-lethal concentration of **CAAQ** (0.25 ppm and 1 ppm); (**D**) egg masses laid by *B. alexandrina*-infected snails exposed to sub-lethal concentration of **CAAQ** (0.25 ppm and 1 ppm); (**E**) egg masses laid by *B. alexandrina* snails exposed to sub-lethal concentration of **CAAQ** (0.25 ppm and 1 ppm). Gelatinous matrix (gm), eggs (e), nuclei (*n*) and micro nucleus (mn). Scale bar: 0.1 mm.

The results showed that **CAAQ** caused some abnormalities in the eggs of *B. alexandrina* Figure 6. The percentage of normal eggs of the exposed snails to 0.25 ppm and 1 ppm were 40.4% and 79.4%, respectively, and of the infected-exposed snails to 0.25 ppm and 1 ppm were 42% and 73.7%, respectively, when compared to 94% and 84% of the control and the infected control groups (*p* ≥ 0.05), respectively, after 24 h of exposure. The percentage of anucleated eggs of the exposed snails to 0.25 ppm and 1 ppm were 200% and 0%, respectively, and of the infected-exposed snails to 0.25 ppm and 1 ppm were 72% and 90%, respectively, when compared to 1% and 5.2% of the control and the infected control groups (*p* ≥ 0.05), respectively, after 24 h of exposure. The percentage of multi-nucleated eggs of the exposed snails to 0.25 ppm and 1 ppm were 46% and 0%, respectively, and of the infected-exposed snails to 0.25 ppm and 1 ppm were 100% and 0%, respectively, when compared to 1% and 15% of the control and the infected control groups (*p* ≥ 0.05), respectively, after 24 h of exposure, Table 3.

The percentage of normal eggs of the exposed snails to 0.25 ppm and 1 ppm were 4% and 18%, respectively, and of the infected-exposed snails to 0.25 ppm and 1 ppm were 5% and 3%, respectively, when compared to 89% and 86.4% of the control and the infected control groups, respectively, after the first week of exposure (*p* ≤ 0.002). The percentage of anucleated eggs of the exposed snails to 0.25 ppm and 1 ppm were 7% and 12%, respectively, and of the infected-exposed snails to 0.25 ppm and 1 ppm were 17% and 4%, respectively, when compared to 7.7% and 9.3% of the control and the infected control groups, respectively, after the first week of exposure (*p* ≤ 0.002). The percentage of multi-nucleated eggs of the exposed snails to 0.25 ppm and 1 ppm were 0% and 8%, respectively, and of the infected-exposed snails to 0.25 ppm and 1 ppm were 0% and 2%, respectively, when compared to 3.4% and 4.3% of the control and the infected control groups, respectively, after the first week of exposure (*p* ≤ 0.002). 

The percentage of normal eggs of the exposed snails to 0.25 ppm and 1 ppm were 5% and 2%, respectively, and of the infected-exposed snails to 0.25 ppm and 1 ppm were 6% and 4%, respectively, when compared to 96.3% and 95.5% of the control and the infected control groups, respectively, after the second week of exposure (*p* ≤ 0.001). The percentage of anucleated eggs of the exposed snails to 0.25 ppm and 1 ppm were 3% and 4%, respectively, and of the infected-exposed snails to 0.25 ppm and 1 ppm were 4% and 17%, respectively, when compared to 2.2% and 2.6% of the control and the infected control groups, respectively, after the second week of exposure (*p* ≤ 0.001). The percentage of multi-nucleated eggs of the exposed snails to 0.25 ppm and 1 ppm were 0% and 9%, respectively, and of the infected-exposed snails to 0.25 ppm and 1 ppm were 0% and 0%, respectively, when compared to 1.5% and 2% of the control and the infected control groups, respectively, after the second week of exposure (*p* ≤ 0.001).

#### 3.3.4. Effect of **CAAQ** on Eggs’ Hatchability of *B. alexandrina* In Vitro

**CAAQ** exposure caused no hatchability at both concentrations of 0.25 and 1 ppm when compared to the control group. Meanwhile, the control hatched after 7 days after the beginning of the experiment and the mean number was 123 ± 11.5.

#### 3.3.5. Scanning Electron Microscopy (SEM) of *S. mansoni* Cercariae

**Figure 7:**
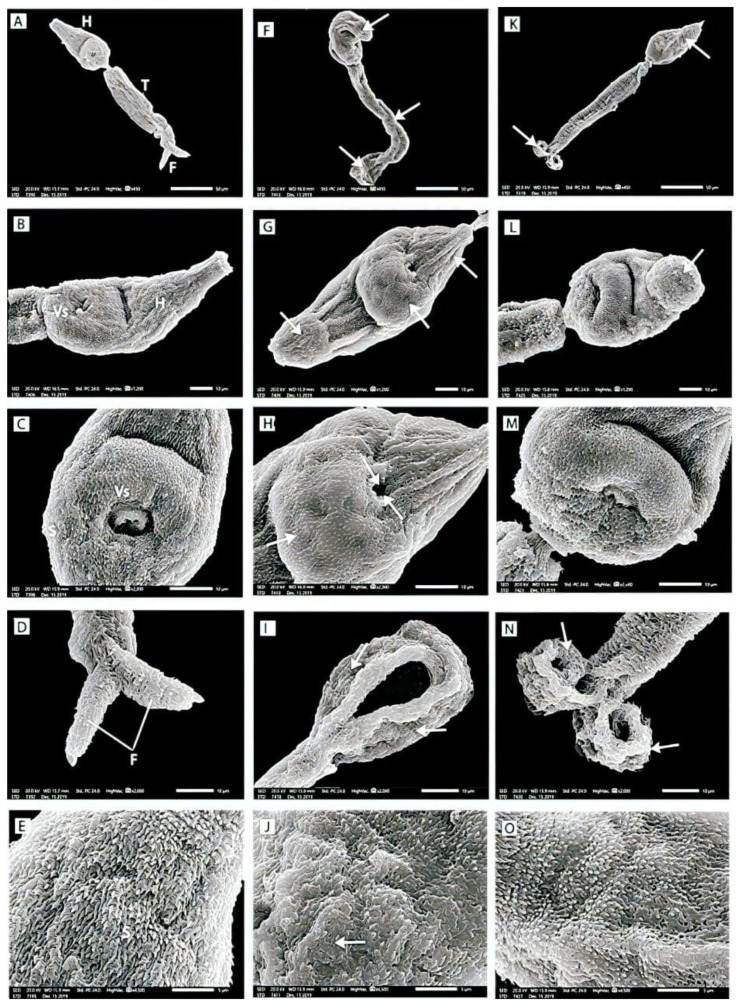
Scanning electron microscopy (SEM) of *S. mansoni* cercariae: (**A**) control cercariae (×450); (**B**) ventral sucker; (**C**) ventral sucker covered with spines; (**D**) tail with its two furculae; (**E**) spines; (**F**) cercaria exposed to AEAA showing body of cercaria (×450); (**G**,**H**) cercaria body (×1200 and ×2300, respectively); (**I**) cercaria tail (×2000); (**J**) cercaria body (×4500); (**K**) cercaria exposed to CAAQ (×450); (**L**) cercaria mouth and body (×1200); (**M**) ventral suckers and mouth (×2300); (**N**,**O**) cercaria tail (×2000 and ×4500, respectively). Head (H), tail (T), furculae (F), ventral sucker (Vs), spines (S) and deformations (arrows).

The cercariae of *S. mansoni* consist of an oval body or head and a long cylindrical tail that is divided into two furculae at the posterior extremity, Figure 7A. The body is covered with several spines, which are posteriorly directed spiny tegmental folds covering the anterior part of the head, Figure 7B. The ventral sucker is well developed and covered with numerous sharp spines directed backwardly, Figure 7C,E. The tail of cercariae and its furculae are covered with spines, which are visible, larger and sharper than those of the body, Figure 7D. 

In the present study, *S. mansoni* cercariae exposed to 50 ppm of **AEAA** (the best compound effect on cercariae) and 50 ppm of **CAAQ** (the best compound effect on snails) showed different morphological changes. 

When *S. mansoni* cercariae were treated by **AEAA**, it caused the whole body of cercariae to become thinner, there was a cut in the tail and the head was swelled and coiled and had terminal deformation, Figure 7F. Body lengths were normal; there were longitudinal wrinkles under and above the ventral sucker and swelling appeared in the mouth area and oral sucker, Figure 7G. Decrease of spines in the outer surface, construction in the area of the ventral sucker, longitudinal wrinkles were observed and the outer surface became peeled, Figure 7H. The tail was extremely attached and formed an enclosed space, Figure 6I. Spines were almost completely peeled, Figure 6J.

**CAAQ** on *S. mansoni* cercariae caused the body to be thinner than the control, forced tail had internal distortion and some foci of peeled spines covered the tail, Figure 7K. There was swelling in mouth and shrinkage in the body’s full length, Figure 7L. There was a swelling area above the ventral sucker toward the mouth end, Figure 7M. The tail was twisted internally and had a wrinkled outer surface, Figure 7N. Some foci of peeled spines covered the tail, Figure 7O.

## 4. Discussion

Our study on structural modification of naturally based heteroarenes such as quinoline, acridine and neocryptolepine to evaluate the biological activity is continued. Diamine linkers with two and three carbon spacers were applied on C-4 position for quinoline core, C-9 position for acridine and C-11 position for neocryptolepine core, followed by formation of ammonium chloride salt, which resulted in the compounds under investigation: **CAAQ**; **AEAA**; **APACQ**; **AEAN**; **APAN** and **APAA**. Modeling studies were performed to investigate the affinity of the prepared compounds to cysteine protease. Proteases are enzymes that are crucial for a variety of biological processes as they catalyze the hydrolysis of peptide bonds. Cysteine proteases are crucial virulence factors in the development of parasite illness and are connected to numerous cellular processes. They are divided into 72 different families. The clan CA papain-family enzymes are the most prevalent and well-characterized CPs in these organisms; as a result, these enzymes have emerged as interesting targets for antiparasitic medications [41,42]. Accordingly, the affinity of the prepared compounds to a target from the papain family was investigated. Protein crystal structure was downloaded from protein data bank, pdb code: 2P7U. All the investigated compounds showed affinity to the selected protein among which compounds **AEAN** and **APAN** recorded the top results.

Biological evaluation demonstrated that **CAAQ** was toxic to adult *B. alexandrina* snails. The sub-lethal concentrations 0.25 ppm and 1 ppm of **CAAQ** were tested on adult *B. alexandrina* snails during the experimental period (2 weeks). The molluscicidal activity of **CAAQ** may be due to the quinoline core which was confirmed important for antischistosomal agents. The ring skeleton and the basic side chain are considered critical for the antischistosomal effect, as the activity was significantly increased by the introduction of the amino side chain into the neocryptolepine core [25,26]. Moreover, introducing amino-alkyl-amino and chloro-side chain was reported to initiate both schistosomicidal and molluscicidal activities in derivatives of neocryptolepines [25,26]. Similar results were obtained by Wright [43] who reported some cryptolepine derivatives that had in vitro and in vivo antimalarial activities. Structurally similar compounds bearing carbazole aminoalcohols were also studied by Wang et al. to prove the contribution of the carbazole core, the amine side chain and stereochemistry to antischistosomal and antiplasmodium activities [24]. **CAAQ** is an alkaloid compound and this may be responsible for its molluscicidal activity. Ibrahim and Abdel-Tawab [44] suggested that the molluscicidal potency of *C. barbata*, the marine algae against *B. alexandrina* snails, may be due to its content of alkaloids, saponins and flavonoids. This finding was in harmony with El-Atti et al. [4] revealing that alkaloids, tannins, flavonoids, terpenoids, saponins and phenolic derivatives presented in Ginger (*Zingiber officinale*) caused its molluscicidal activity against *M. cartusiana* snails. Moreover, the methanolic extract of *N. oleander* and *T. stans* plants was reported to have molluscicidal activity against *B. alexandrina* snails, and [5] revealed that *N. oleander* had higher activity than *T. stans* methanolic extracts which may be due to the higher concentrations of the secondary metabolites like alkaloids, saponins, steroids, tannins, terpenoids, anthraquinones and coumarins found in *N. oleander* [5]. 

In this study, **CAAQ** showed a larvicidal activity against miracidia and cercariae after 90 min of exposure. The miracicidal potency was much higher than the cercaricidal activity. The obtained results showed that miracidia are more tolerant to the toxic effect of the tested compound than cercariae. This may depend on the presence of heavy ciliated tegument in miracidia that could cause reduction in the toxic effect of the tested materials [45,46]. Similar results were obtained by Sheir et al. [7] who showed the effect of peels of *Citrus limon* against *S. mansoni* miracidia and cercariae and suggested that its larvicidal activity may be due to the presence of the limonene. These results may be due to the tested compound being alkaloid in nature. It has been considered that alkaloids were responsible for the cytotoxic effects as they were approved for their activity against cercariae and miracidia. The present finding is in accordance with that reported by Membe Femoe et al. [6] who studied the effect of the plants *Sida acuta* (HESa) and *Sida rhombifolia* (HESr) hydroethanolic extracts against *Schistosoma mansoni* cercariae. They reported that the cercaricidal potential of these ingredients as their phytochemicals belonging to alkaloids might damage the cercariae tegument and disturb its motor activity. In addition, Dos Santos et al. [47] found that diethyl 4- phenyl-2, 6-dimethyl-3, 5 pyridine dicarboxylate, a penta-substituted pyridine alkaloid from the rhizome of Jatropha elliptica, had strong cercaricidal and miracicidal activities. The roots of this plant showed molluscicidal activity and contain alkaloid terpenoids, coumarin, lignoid and steroids. Njogu et al. [48] reported that four furoquinoline alkaloids were isolated from the plant *Teclea nobilis* which showed antimiracidial activity. Also, Ibrahim et al. [5] stated both the plants *N. oleander* and *T. stans* extracts had a cercaricidal activity. The authors mentioned that *N. oleander* extract was more toxic than *T. stans* extract on cercariae and they suggested this difference in the mortality may be due to the presence of more alkaloids, saponins and flavonoids in *N. oleander* extract.

In the present study results, SEM of *S. mansoni* cercariae exposed to the tested compounds (50 ppm of **AEAA** and **CAAQ**) showed different morphological changes. The present results were consistent with the report of Eissa et al. [49] who showed that cercariae exposed to miltefosine drug showed partial detachment of the body from the tail, and in some cercariae, the body was completely separated from the tail and had the appearance of surface blebbing with focal loss of spines from the tegument. Membe Femoe et al. [6] reported that the phytochemicals such as alkaloids, flavonoids, polyphenols, coumarins and steroids could damage the cercarial tegument and disturb the motor activity of cercariae. In addition, surface blebbing is considered as a stress indicator as discussed by Manneck et al. [50]. Zhang and Coultas [51] showed that two plant-derived compounds, plumbagin and sanguinarine, caused morphological alterations and tegumental changes on the worms of *S. mansoni* treated by the two compounds. They concluded this tegumental damage might be due to the surface of the parasite’s membrane and its tegumental structure which could control the immune system modulation, evasion and nutrient uptake, which has an effect on the worm survival in the host. Meshnick and coworkers [52] and Xiao et al. [53] declared that the treatment with artemether caused intensive tegumental alterations. That may be due to the interaction between artemisinin and intra-parasitic heme (iron) that could be a role to activate artemisinin inside the parasite into toxic free radicals.

In the current study, exposure of the snails to **CAAQ** at sub-lethal concentrations led to a remarkable reduction in egg production. This reduction of egg laying may be related partially to their high mortality rates and different periods of ceasing egg laying. The low values of egg-laying capacity could be attributed to the molluscicide effect of the plant, which is proved to cause a significant impact on the egg-laying production [44,54]. These results coincide with that described by Rizk et al. [55] who found that Chloroform extract of the medicinal plant *Haplophyllum tuberculatum* (family Rutaceae) against *B. alexandrina* snails recorded reduction in egg-laying capacity. They suggested that reduction was due to disturbance in egg-laying enzyme (phenol oxidase). Abdel Salam et al. [56] proved that sub-lethal concentration of thymol (plant molluscicide) for 4 weeks showed an oviposition inhibitory activity and reproduction rate against *B. alexandrina* snails.

In the current work, *B. alexandrina* snails treated with sub-lethal concentrations of **CAAQ** increased the abnormal egg masses that were laid by the exposed snails. These observations of abnormal egg masses could be the consequences of the inhibiting action of the experimental compound on the pathways that control oogenesis, laying and eggs’ formation in the genital tract. Natural molluscicides (*Agave filifera* and *A. attenuate*) caused a significant increase in the percentage of abnormal laid eggs of *B. alexandrina* snails as mentioned by [57]. *B. alexandrina* snails treated with *Euphorbia lactea* (plant extract) caused a significant increase in the number of abnormal laid egg masses [58]. Demetillo et al. [59] reported that treatment with crude leaf extract of *Cymbopogon citratus* (lemon grass) against *Pomacea canaliculata* (golden apple snail) caused a significant alteration in the normal development of the eggs as a high percentage of eggs were with empty embryos. They suggested that the presence of saponin and flavonoids in *C. citratus* might stop the development of the embryo of the apple snail and prevent hatching. Ibrahim and Abdel-Tawab [44] stated that the alterations in the embryonic development (embryos died and some degenerated) of *B. alexandrina* snail eggs exposed to the ethanolic extract of *Cystoseira barbata*, marine algae, may be due to the higher concentration adversely affected hatchability percentages of these eggs.

Concerning the effect of sub-lethal concentrations of **CAAQ** on the hatchability of *B. alexandrina* eggs, the tested compound caused no hatchability when compared to the control snails. Many investigators proved a relationship between metabolic disorders of exposed snails to different plant molluscicides and the size and viability of their egg masses [55,56]. Hamdi and Rawi [57] showed the effect of *Agave filifera* and *A. attenenuate* plants (family: Agavaceae) on *B. alexandrina* snails, causing inhibition of egg production, a significant increase in the abnormality percentage of eggs and a decrease in their hatchability of the same batch of eggs. Mostafa and Gawish [60] reported that the algal culture filtrate of micro-alga (*Spirulina platensis*) caused a significant decrease in the hatchability of eggs of treated *B. alexandrina* snails due to the presence of alkaloids, saponins and mineral salts. Osman et al. [61] showed that Mirazid caused impairment in the hatchability of *B. alexandrina* eggs. The present results were in accordance with those obtained by Fatimatuzzahra et al. [62] who explained the inhibition in hatchability of the tree sorrel extract (*Averrhoa bilimbi* L.) and snake plant as a natural ovicidal compound of the apple snail (*Pomacea canaliculata* L.). This could be due to the alkaloids, flavonoids, saponins, terpenoids and tannins in the plants. 

## 5. Conclusions

Six *N*-heterocycles analogues based on natural quinoline, acridine and neocryptolepine core structures were synthesized and tested as molluscicides. Molecular docking studies were performed to investigate the affinity of the prepared compounds for cysteine protease protein. All the tested compounds showed promising affinities to cysteine protease. 7-Chloro 4-amino ethyl amino quinoline hydrochloride salt **CAAQ** was the most effective compound against *B. alexandrina* snails with lethal concentrations, LC_50_ and LC_90_ values of 171.95 and 335.79 ppm, respectively. N1-(5-methyl-5H-indolo [2, 3-b] quinolin-11-yl) propane-1,3-diamine **APAN** had the most efficiency against miracidia with LC_50_ and LC_90_ values of 3.36 and 0.65 ppm, respectively. Meanwhile, N1 (acridin-9-yl) ethane 1,2 diamine **AEAA** was the most effective against cercariae with LC_50_ and LC_90_ values of 1.25 and 0.22 ppm, respectively. Based on the aforementioned evaluations, the six prepared heterocycles analogues are considered promising as molluscicidal and larvicidal entities.

## Figures and Tables

**Table 1:** Docking results for the synthesized compound in cysteine protease PDB code: 2P7U.

PDB Code	Compound	S	RMSD	Amino Acids and Type of Bonding
2P7U	**CAAQ**	−4.8697	1.8618	ASP117 (H-donor), ASP117 (ionic)
	**AEAN**	−5.9386	1.4522	MET68 (H-donor), MET68 (H-donor), CYS25 (H-donor), ASP161 (H-donor), ALA208 (*Pi*-H)
	**APAN**	−5.6194	1.7254	ASP117 (H-donor), ASP117 (ionic), LEU67 (*Pi*-H)
	**AEAA**	−5.2061	2.0628	CYS25 (H-donor), GLY66 (H-donor), MET68 (H-donor)
	**APAA**	−5.4576	1.5371	ASP117 (H-donor), ASP117 (ionic)
	**APACQ**	−5.2330	1.7619	ASP117 (H-donor), ASP117 (ionic), LEU67 (*Pi*-H)
	**D1R**	−5.678	1.4811	GLY66 (H-donor), GLY66 (H-acceptor), ASP161 (H-donor), GLN19 (H-acceptor), TRP184 (H-acceptor)

**Table 2:** Effect of sub-lethal concentrations of CAAQ on the mean number of egg masses/snail/week of *B. alexandrina* snails.

Time	Exposure	Non-Infected Snails	Infected Snails
24 h	Control	11.0 ± 0.05	0.18 ± 0.08
	0.25 ppm	0.04 ± 0.05	0.08 ± 0.02
	1 ppm	0.07± 0.09	0.18 ± 0.02
1st week	Control	2.80 ± 0.38	1.97 ± 0.46
	0.25 ppm	0.16 ± 0.02 *	0.16 ± 0.02 *
	1 ppm	0.4 ± 0.28 *	0.09 ± 0.08 *
2nd week	Control	2.67 ± 0.33	1.96 ± 0.47
	0.25 ppm	0.17 ± 0.04 *	0.18 ± 0.05 *
	1 ppm	0.06 0.05 *	0.09 ± 0.12 *

Data were expressed as mean ± SD. * Significant difference at *p* ≤ 0.05, when compared to the control group.

**Table 3:** Effect of sub-lethal concentrations of **CAAQ** on the egg abnormalities of *B. alexandrina* snails.

Time	Treatments	Non-Infected Snails			Infected Snails		
		Normal	Anucleated	Multi-Nucleated	Normal	Anucleated	Multi-Nucleated
24 h	control	94	1	1	84	5.2	15
	0.25 ppm	40.4	200	46	42	72	100
	1 ppm	79.4	0	0	73.7	90	0
1st week	control	89	7.7	3.4	86.4	9.3	4.3
	0.25 ppm	4	7	0	5	17	0
	1 ppm	18	12	8	3	4	2
2nd week	control	96.3	2.2	1.5	95.5	2.6	2
	0.25 ppm	5	3	0	6	4	1
	1 ppm	2	4	9	4	17	0

Data were expressed as percentage (%).

## Data Availability

Not applicable.

## Supplementary Materials

The following supporting information can be downloaded at: https://www.mdpi.com/article/10.3390/pharmaceutics15041200/s1, ^1^H NMR and ^13^C NMR for the synthesized compounds were presented in supplementary materials.
