# Supplementary material for: Molluscicidal and Larvicidal Potency of N-Heterocylic Analogs against Biomophalaria alexandrina Snails and Schistosoma mansoni Larval Stages
Source: Pharmaceutics. 2023 Apr 10;15(4):1200. doi: 10.3390/pharmaceutics15041200 (PMC10142358; doi:10.3390/pharmaceutics15041200)
Supplement: Supplementary file 1 [file pharmaceutics-15-01200-s001.zip › pharmaceutics-2257479-supplementary.pdf]

Supplementary Materials

Molluscicidal and Larvicidal Potency of *N*-Heterocylic Analogs against *Biomophalaria alexandrina* Snails and *Schistosoma mansoni* Larval Stages

Sherin K. Sheir [1]; Elshaymaa. Elmongy [2]*; Azza H. Mohamad [1]; Gamalat Y. Osman[1]; Shimaa E. Bendary [1]; Abdullah A. S. Ahmed [3]; Reem Binsuwaidan[2] and Ibrahim El-Tantawy El-Sayed[3]

[1] Zoology Department, Faculty of Science, Menoufia University, Shibin El Kom 32511, Egypt; sherin.sheir@yahoo.com (S.K.S.); azza_hassan_2006@yahoo.com (A.H.M.); dr.gyosman@yahoo.com (G.Y.O.); samshosalania52@yahoo.com (S.E.B.)
[2] Department of Pharmaceutical Sciences, College of Pharmacy, Princess Nourah bint Abdulrahman University, P.O. Box 84428, Riyadh 11671, Saudi Arabia; rabinsuwaidan@pnu.edu.sa
[3] Chemistry Department, Faculty of Science, Menoufia University Shibin El-Kom, Menoufia 32511, Egypt; chemist_abdullah_2009@yahoo.com (A.A.S.A.); ibrahimtantawy@yahoo.co.uk (I.E.-T.E.-S.)
* Correspondence: eielmongy@pnu.edu.sa

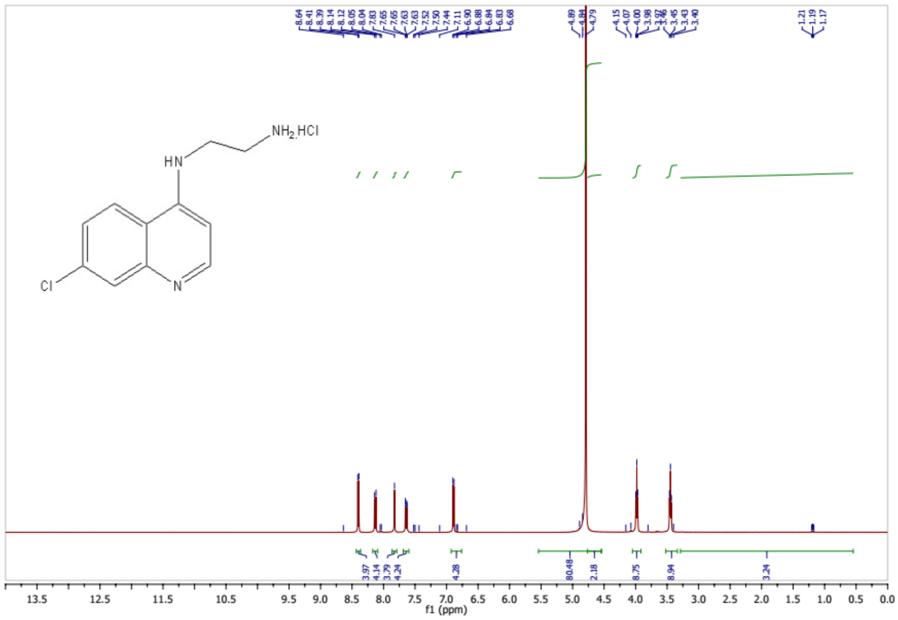

^{1}H NMR of **4a** in D_2O

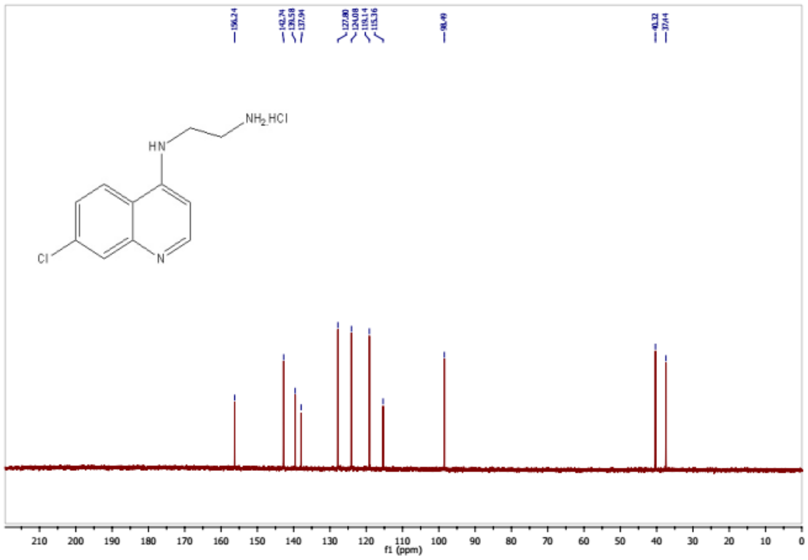

^{13}C NMR of **4a** in D_2O

Synthesis of *N1*-(7-Chloroquinolin-4-yl)-propane-1,3-diamine hydrochloride **4b**

An excessive amount of 1,3 diamino propane **2b** was added to 4,7-dichloroquinoline **1a** under reflux condition for 2 hrs. to obtain free amine product 3 with good yield. The mixture was poured into ice water, filtered off and dried to afford the free amine **3b**. In the next step 1M of hydrochloric acid was added to the ethanolic solution of free amine **3b** and the pH was adjusted till the formation of the hydrochloride salt of free amine **4b**

N^1-(7-Chloroquinolin-4-yl)-propane-1,3-diamine hydrochloride **4b**: Yellowish white solid, yield: 86% ^{1}H NMR (D_2O, 400 MHz) δ (ppm): 8.47 (m,1H, CH_{Ar}), 7.92 (m, 1H, CH_{Ar}), 7.71 (m, 1H, CH_{Ar}), 7.37 (1H, br s, NH), 7.28 (m,1H, CH_{Ar}), 6.34 (m, 1H, CH_{Ar}), 3.47–3.28 (m, 2H, CH_2), 3.11–3.02 (m, 2H CH_2), 2.74 (2H, br s, NH_2), 1.97–1.93 (2H, m, CH_2); ^{13}C NMR (D_2O, 100 MHz,) δ (ppm): 155.9, 144.4, 140.3, 138.2, 127.2, 126.4, 120.4, 117.3, 98.7, 42.2, 40.6, 30.6.

Synthesis of aminoalkylamino acridine hydrochloride (**4c**,**d**)

To 9-chloroacridine **1b** (0.12 g, 1 mmol) was added an excess amount of ethylene diamine **2a** or 1,3 di amino propane **2b** (3 mmol); then, the reaction mixture was refluxed for 1–2 h, until the complete consumption of the starting materials occurred as monitored by TLC. The mixture was cooled and poured into ice water, filtered off and dried to yield the desired products **3c**, **d**. further addition of 1M hydrochloric acid to ethanolic solution of free amine **3c, d** with adjustment of pH till the formation of the hydrochloride salt of free amines **4c, d.**

N1-(acridin-9-yl)ethane-1,2-diamine hydrochloride **4c**: Yellow solid, yield: 63%, ^{1}H NMR (D_2O, 400 MHz) δ: 8.40–7.18 (m, 8H, CH_{Ar}), 3.97 (br.s, 2H,CH_2), 3.02 (m, 2H, CH_2). ^{13}C NMR (D_2O,100 MHz) δ: 153.3, 149.2, 131.6, 130.0, 126.5, 123.8, 121.6, 46.2, 40.5.

N1-(acridin-9-yl)propane-1,3-diamine hydrochloride **4d**: Yellow solid, yield: 60%, ^{1}H NMR (D_2O, 400 MHz), δ ppm: 11.62(s, 1H, NH), 11.45 (s, 2H, NH_2), 8.00–7.42 (m, 8H, CH_{Ar}), 3.64 (br.s, 2H, CH_2), 2.42 (m, 2H, CH_2), 1.61 (m, 2H, CH_2). ^{13}C NMR (D_2O,100 MHz) δ:161.5,142.3, 129.7, 127.8, 126.3, 120.8, 114.8, 41.3, 39.4, 31.5.

The general procedure for the synthesis of 11-aminoalkylamino neocryptolepine hydrochloride **7a,b**

To 11-chloroneocryptolepine **5** (1 g, 3.75 mmol) was added in excess amount ethylene diamine **2a** or 1.3-diaminopropane **2b** (11.26 mmol) in DMF (1 mL) and (1.57 mL,11.26 mmol) of triethylamine as a base. The mixture was refluxed for 2 h. and cooled to room temperature, then poured into ice water; the precipitated products **6a, b** were filtered off, washed with water, In the next step 1M of hydrochloric acid was added to the ethanolic solution of free amines with adjustment of pH till the formation of the hydrochloride salt of free amines **7a, b**

N1-(5-methyl-5H-indolo[2,3-b]quinolin-11-yl)ethane-1,2-diamine hydrochloride**7a**: Yellowish-orange solids; yield: 76%; ^{1}H-NMR (D_2O, 400 MHz) δ: 8.10 (d, J = 2.2 Hz, CH_{Ar}), 8.08–8.05 (m, 1H, CH_{Ar}), 7.77–7.74 (m, 1H, CH_{Ar}), 7.60 (m, 1H, CH_{Ar}), 7.53 (m, 1H, CH_{Ar}), 7.45 (m, 1H, CH_{Ar}), 7.22–7.18 (m, 1H, CH_{Ar}), 6.35 (s, 1H, CH_{Ar}), 4.18 (s, 3H, *N*-CH_3), 3.74 (m, 2H, CH_2), 2.98–2.95 (m, 2H, CH_2), 1.51 (s, 2H, CH_2).

N1-(5-Methyl-5H-indolo[2,3-b]quinolin-11-yl)propane-1,3-diamine hydrochloride **7b:** Yellow solid yield: 70%, ^{1}H-NMR (D_2O, 400 MHz) ppm δ: 7.15–7.98 (m, 8H, CH_{Ar}) , 4.24 (s, 3H, *N*-CH_3), 4.07 (m, 2H, CH_2) , 3.06 (t, 2H, CH_2, *J* = 8 Hz), 1.85 (t, 2H, CH_2, *J* = 8 Hz).

^{13}C NMR (D_2O, 100 MHz) ppm δ: 152.1, 136.1, 130.4, 125.7, 124.1, 121.4, 120.3, 114.7, 49.4, 41.5, 32.9, 29.1.
